# Upregulated MicroRNA-29a by Hepatitis B Virus X Protein Enhances Hepatoma Cell Migration by Targeting PTEN in Cell Culture Model

**DOI:** 10.1371/journal.pone.0019518

**Published:** 2011-05-05

**Authors:** Guangyao Kong, Junping Zhang, Shuai Zhang, Changliang Shan, Lihong Ye, Xiaodong Zhang

**Affiliations:** 1 Key Laboratory of Molecular Microbiology and Technology of Ministry of Education, Department of Cancer Research, Institute for Molecular Biology, College of Life Sciences, Nankai University, Tianjin, People's Republic of China; 2 Department of Biochemistry, College of Life Sciences, Nankai University, Tianjin, People's Republic of China; French National Center for Scientific Research - Institut de biologie moléculaire et cellulaire, France

## Abstract

Hepatitis B virus X protein (HBx) plays important roles in the development of hepatocellular carcinoma (HCC). MicroRNAs (miRNAs) contribute to cancer development by acting as oncogenes or tumor suppressors. Previously, we reported that HBx was able to promote the migration of hepatoma HepG2 cells. However, the regulation of miRNAs in the development of HBV-related HCC is poorly understood. In the present study, we reported that miR-29a was a novel regulator of migration of hepatoma cells mediated by HBx. Our data showed that the expression of miR-29a was dramatically increased in *p21-HBx* transgenic mice, HBx-transfected hepatoma HepG2-X (or H7402-X) cells and HepG2.2.15 cells that constitutively replicate HBV. However, our data showed that miR-29a was upregulated in 4 of the 11 clinical HCC samples. We found that the overexpression of miR-29a promoted the migration of HepG2 cells, while a specific miR-29a inhibitor could partially abolish the enhanced migration of HepG2-X cells. Moreover, we identified PTEN was one of the target genes of miR-29a in HepG2 cells. The deletion of the miR-29a-binding site was able to abolish the role of miR-29a in suppression of luciferase activity of the PTEN 3′UTR reporter. Meanwhile, the overexpression of PTEN was able to reverse the promoted migration of HepG2 cells mediated by miR-29a. Moreover, our data showed that the modulation of Akt phosphorylation, a downstream factor of PTEN, was involved in the cell migration enhanced by miR-29a, suggesting that miR-29a is responsible for the cell migration through its target gene PTEN. Thus, we conclude that miR-29a is involved in the regulation of migration of hepatoma cells mediated by HBx through PTEN in cell culture model.

## Introduction

Hepatocellular carcinoma (HCC) is one of the most common malignant tumors in the world. Among the well known risk factors for HCC, chronic infection with hepatitis B (HBV) or C (HCV) virus is present in more than 85% of primary liver cancers [1]. The HBV X protein (HBx), an essential factor for HBV replication, is thought to play a key role in the molecular pathogenesis of HBV-related HCC (HBV-HCC) [2]. Previous study revealed that HBx knocked into the p21 locus caused hepatocellular carcinoma in mice [3]. Our laboratory has focused on the investigation of hepatocarcinogenesis mediated by HBx. Our and other reports have demonstrated that HBx is able to promote migration and invasion of hepatoma cells by upregulation of osteopontin, Capn4, matrix metalloproteinases, MIG, and deregulation of intercellular adhesion [4], [5], [6], [7]. However, a comprehensive understanding of the underlying mechanism by which HBx promotes migration needs further elucidation.

MicroRNAs (miRNAs) are evolutionary conserved small RNAs affecting gene expression at the posttranscriptional level through translational repression and/or target messenger RNAs degradation in a sequence-dependent manner [8]. Recent studies have revealed that miRNAs participate in many cellular processes including proliferation, development, differentiation, or even in tumorigenesis [9]. Alterations of the expression patterns of miRNAs have been found in different human tumors [10]. Despite the growing evidence for their importance in carcinogenesis, limited information is available about their function in HBV-HCC. Previously, miR-29a was implicated in chronic lymphocytic leukaemia, cholangio carcinoma and lung cancer by deregulation of its target gene Tcl1 and DNMT3 as a tumor suppressor [11], [12]. However, it is also reported that miR-29a promote tumorigenesis in breast cancer and acute myeloid leukemia [13], [14], and recently, Santanam U et al. reported that overexpressing miR-29 in mouse B cells contributes to B-cell chronic lymphocytic leukemia in transgenic mouse model [15]. These studies suggest a context-dependent pattern for miR-29a in tumorigenicity.

Phosphatase and tensin homolog (PTEN) is a protein and phosphoinositide phosphatase which is originally identified as a tumor suppressor frequently mutated or deleted in various human cancers to promote tumorigenesis [16], [17]. Interestingly, accumulating evidence indicates that deregulated PTEN expression in hepatocytes, rather than PTEN mutations or deletions, represents a critical factor in the development of HCC. It has been reported that PTEN is downregulated in HCC patients by immunohistochemistry assay [18]. PTEN was indicated to be able to inhibit migration through regulation of PI3K/Akt pathway or SRC family kinases [19], [20]. PTEN was also shown to be a direct target of miR-21 and miR-221& 222, and contribute to cell migration [21], [22]. However, whether other miRNAs are also involved in the regulation of PTEN remains unclear.

In the present study, we sought to gain insight into the regulation of miR-29a in HBV-HCC. Our finding shows that miR-29a is able to directly regulate PTEN in the promotion of hepatoma cell migration mediated by HBx. Our data provide new insights into the mechanism of promotion of hepatoma cell migration induced by HBx.

## Results

### MiR-29a is upregulated in the *p21-HBx* transgenic mice and stable HBx-transfected hepatoma cells

**Figure 1:**
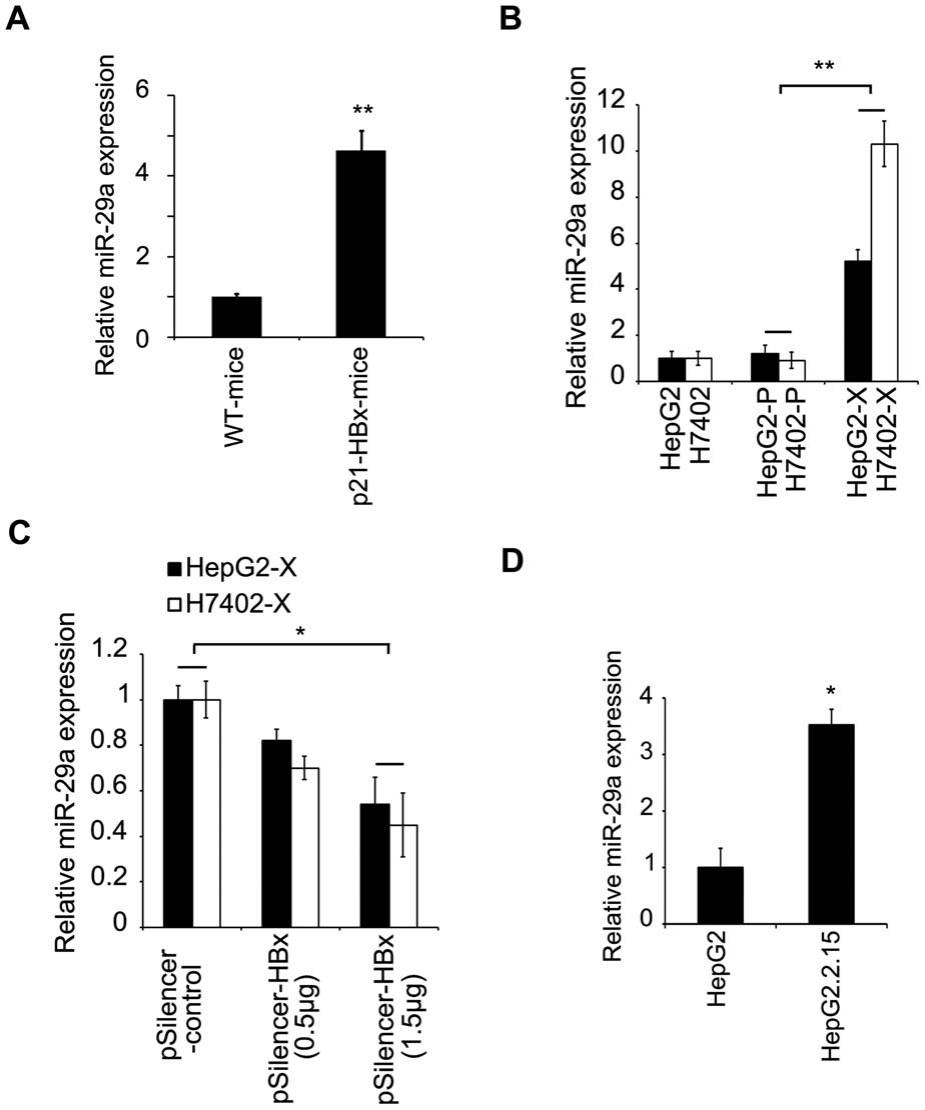
HBx upregulates miR-29a. (A) The relative expression of miR-29a for 6-month-old *p21-HBx* transgenic mice versus WT mice was detected by qRT-PCR. (B) The expression of miR-29a in stable HBx-transfected hepatoma cell lines was examined by qRT-PCR. (C) The expression levels of miR-29a in HepG2-X (or H7402-X) were examined by qRT-PCR after treatment with HBx RNAi in a dose dependent manner. (D) The relative expression level of miR-29a in HepG2.2.15 cells was examined by qRT-PCR. Plotted are the means ± SD of three samples normalized to U6. Statistically significant differences, arbitrarily set to 1.0, are indicated: *P<.05, **P<.01, Student's *t* test.

To gain insight into the biological role of HBx in miRNA expression pattern, we analyzed the expression of miR-29a in *p21-HBx* transgenic mice by quantitative reverse-transcription PCR (qRT-PCR). *HBx* gene knock-in transgenic mouse model (termed *p21-HBx* transgenic mice) was generated by homologous recombination. HBx gene was integrated into the mouse p21 locus. Both male and female *p21-HBx* transgenic mice developed HCC after the age of 18 months [3]. We compared the miR-29a expression of transgenic mouse liver tissues with wild type mice of the same strain (C57BL/6), sex, and age (6 months old) and found that miR-29a was significantly upregulated in the transgenic mice (Fig. 1A). Next, we determined whether miR-29a was expressed differently in human hepatoma cells with stable HBx expression. Previously, we established the engineered cell lines including HepG2-X (or H7402-X) which was stably transfected with HBx, and HepG2-P (or H7402-P) which was stably transfected with an empty pcDNA3.0 vector [23]. Then, we found that miR-29a was upregulated in both HepG2-X and H7402-X cells (Fig. 1B). In order to further verify whether this upregulation correlated with the HBx expression, we also examined the expression of miR-29a after HBx knockdown by RNAi. The results showed that the miR-29a expression was reduced after HBx knockdown in a dose dependent manner (Fig. 1C). Moreover, we showed that the miR-29a expression level was upregulated in HepG2.2.15 cells that constitutively replicate HBV relative to HepG2 cells (Fig. 1D). All the data strongly support that HBx upregulates the expression of miR-29a.

To determine whether miR-29a was expressed differentially in human primary liver cancer tissues, we measured the miR-29a expression levels in 11 pairs of human HCC tissues and peritumor tissues by qRT-PCR (Table S2). The result showed that all clinical HCC tissues and their peritumor tissues were positive for HBx mRNA (Fig. S1A). However, among the 11 HBV-related HCC samples analyzed, the miR-29a levels were only significantly increased in 4 HCC samples in comparison with the adjacent noncancerous hepatic tissues (Fig. S1B). Correlation between expression levels of miR-29a and HBx in tissues was explored using Pearson′s correlation coefficient, but no correlation was observed.

### MiR-29a promotes migration of hepatoma cells

**Figure 2:**
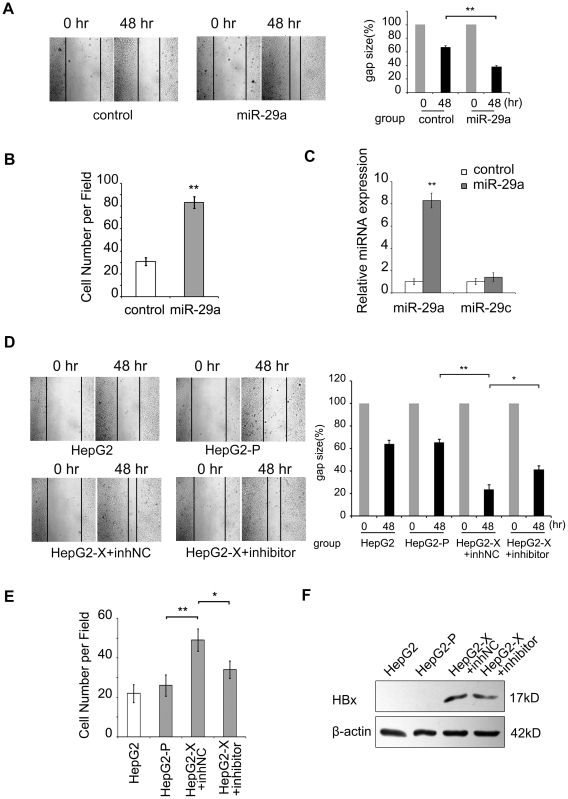
MiR-29a increases the migration of hepatoma cells. (A) A wound healing assay was performed on HepG2 cells (1×10^6^, 6-cm plate) transfected with either an empty vector (control) or a miR-29a expression vector (3 µg). One representative experiment is shown. Black arrows indicate the wound edge. The residual gap between the migrating cells from the opposing wound edge is expressed as a percentage of the initial scraped area. (B) Modified Boyden's chamber assays showed that the cell migration was promoted by the miR-29a in HepG2 cells. A histogram shows the relative cell number of five randomly selected fields. (C) The expression levels of miR-29a and miR-29c were examined by qRT-PCR after overexpression of miR-29a in HepG2 cells. (D) A wound healing assay was performed on HepG2, HepG2-P and HepG2-X cells transfected with a miR-29a inhibitor (100 nM) or inhibitor negative control (inhNC). One representative experiment is shown. Black arrows indicate the wound edge. The residual gap between the migrating cells from the opposing wound edge is expressed as a percentage of the initial scraped area. (E) Modified Boyden's chamber assays showed that the cell migration was blocked by miR-29a inhibitor in HepG2-X cells. A histogram shows the relative cell number of five randomly selected fields. (F) Western blot analysis showed that the expression of HBx protein was not affected by the treatment with miR-29a inhibitor in HepG2-X cells. Statistically significant differences are indicated: *P<.05, **P<.01, Student's *t* test.

We supposed that high expression of miR-29a may be associated with migration capability of hepatoma cells. Therefore, we tested the expression level of miR-29a using the hepatoma cell lines which were reported with different metastasis potential as a model, such as MHCC-97H and MHCC-97L [24]. The data showed that the expression level of miR-29a was positively correlated with the metastasis potential of the cells (Fig. S2A). Then, we assessed the effect of miR-29a on the migration in HepG2 cells by wound healing and modified Boyden's chamber assays. As shown in Fig. 2A and 2B, the overexpression of miR-29a resulted in a remarkable increase in the cell migration ability relative to the control group. The expression level of miR-29a was examined by qRT-PCR after overexpression of miR-29a. The data showed that the expression of miR-29a was significantly increased, while the expression of miR-29c was not affected by the treatment, suggesting that the overexpression of miR-29a is specific (Figure 2C).

To test whether miR-29a regulates the migration of hepatoma cells mediated by HBx, we used a miR-29a specific inhibitor to block miR-29a expression in HepG2-X cells. Interestingly, wound healing and modified Boyden's chamber assays showed a significant reduction of migration of HepG2-X cells after the treatment (Fig. 2D and 2E). Meanwhile, we confirmed that the miR-29a inhibitor did not affect the expression level of HBx protein in HepG2-X cells (Fig. 2F). Thus, our finding reveals that miR-29a is involved in the regulation of migration of hepatoma cells mediated by HBx.

### MiR-29a directly inhibits the expression of PTEN by binding 3′UTR of PTEN mRNA

**Figure 3:**
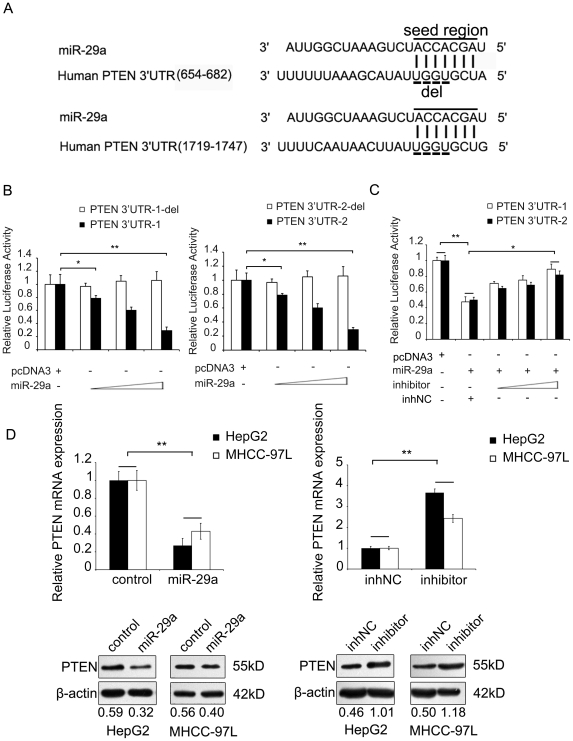
MiR-29a targets PTEN. (A) Sequence alignment between miR-29a and the 3′UTR of human PTEN mRNA. Solid line, seed match region; dashed line, seed-deleted region. (B) Luciferase reporter gene assay showed the effect of miR-29a on the activity of PTEN 3′UTR reporter. Co-transfection was performed using the plasmids, such as human PTEN 3′UTR (PTEN 3′UTR-1 or PTEN 3′UTR-2) or the miR-29a-binding site-deleted (del) PTEN 3′UTR (PTEN 3′UTR-1-del or PTEN 3′UTR-2-del), a miR-29a expression plasmid (50, 100, and 200 ng), in HepG2 cells. Empty pcDNA3.0 plasmid (200 ng) was used as a negative control. (C) Luciferase reporter gene assay showed that the decreased luciferase activities of PTEN 3′UTR reporter (PTEN 3′UTR-1 or PTEN 3′UTR-2) in HepG2 cells mediated by miR-29a overexpression (200 ng) was rescued by a miR-29a inhibitor in a dose dependent manner (30, 50, and 100 nM). (D) Immunoblot and qRT-PCR showed that miR-29a (3 µg, 6-cm plate) induced a decrease in endogenous human PTEN protein and mRNA in HepG2 and MHCC-97L cells, which was increased by transfection with a miR-29a inhibitor. GAPDH and β-actin were used as internal controls. Protein bands were quantified using Quantity One software (Bio-Rad). The value under each lane indicates the relative expression level of the PTEN, which is represented by the intensity ratio between PTEN and β-actin bands in each lane. Statistically significant differences are indicated: *P<.05, **P<.01, Student's *t* test.

To search for the miR-29a target genes, we identified two regions complementary to its seed region in the 3′UTR of human PTEN mRNA using the DIANA microT v3.0 algorithm (Fig. 3A), which was also confirmed by TargetScan and PicTar. To validate whether miR-29a directly recognizes the 3′UTR of PTEN mRNA or not, firstly, we cloned two sequences with the predicted target site of miR-29a (PTEN 3′UTR-1 and PTEN 3′UTR-2) or predicted target site deleted sequences (PTEN 3′UTR-1-del and PTEN 3′UTR-2-del) into downstream of the pGL3-control luciferase reporter gene vector, respectively. When the PTEN 3′UTR wild-type or deletion-type vector was cotransfected with miR-29a, the luciferase activity of the PTEN 3′UTR wild-type vector was significantly decreased compared with the deletion-type vector in a dose-dependent manner (Fig. 3B). Moreover, when both miR-29a and the specific miR-29a inhibitor were cotransfected with the PTEN 3′UTR wild-type vector, the luciferase activity of PTEN 3′UTR wild-type vector was rescued by the inhibitor of miR-29a in a dose dependent manner (Fig. 3C). Furthermore, the overexpression of miR-29a was able to markedly downregulate the expression of endogenous PTEN at the levels of mRNA and protein in HepG2 or MHCC-97L cells. Meanwhile, the miR-29a inhibitor was able to upregulate the expression of PTEN at the levels of mRNA and protein (Fig. 3D). Moreover, the relative expression levels of PTEN were examined by qRT-PCR in 11 clinical HCC tissues. The data showed that the expression level of miR-29a was inversely correlated with the expression level of PTEN (Fig. S2B), suggesting that miR-29a negatively regulates the expression of PTEN by directly targeting the 3′UTR of mRNA.

Previously, it was reported that HBx was able to decrease the expression of PTEN [25]. Western blot analysis confirmed that PTEN was downregulated in HepG2-X (or H7402-X) cells relative to controls (Fig. S2C). While, knockdown HBx by RNAi could increase the expression of PTEN in HepG2-X (or H7402-X) cells in a dose dependent manner (Fig. S2D). Thus, our data are consistent with the reports that HBx downregulates the expression of PTEN. As expected, we showed that the levels of PTEN were negatively correlated with the metastasis potential in a model of MHCC-97H cells/MHCC-97L cells (Fig. S2E), suggesting that PTEN is negatively associated with the expression of miR-29a. It supports that PTEN is a target gene of miR-29a.

### PTEN is involved in the enhanced migration of hepatoma cells mediated by miR-29a

**Figure 4:**
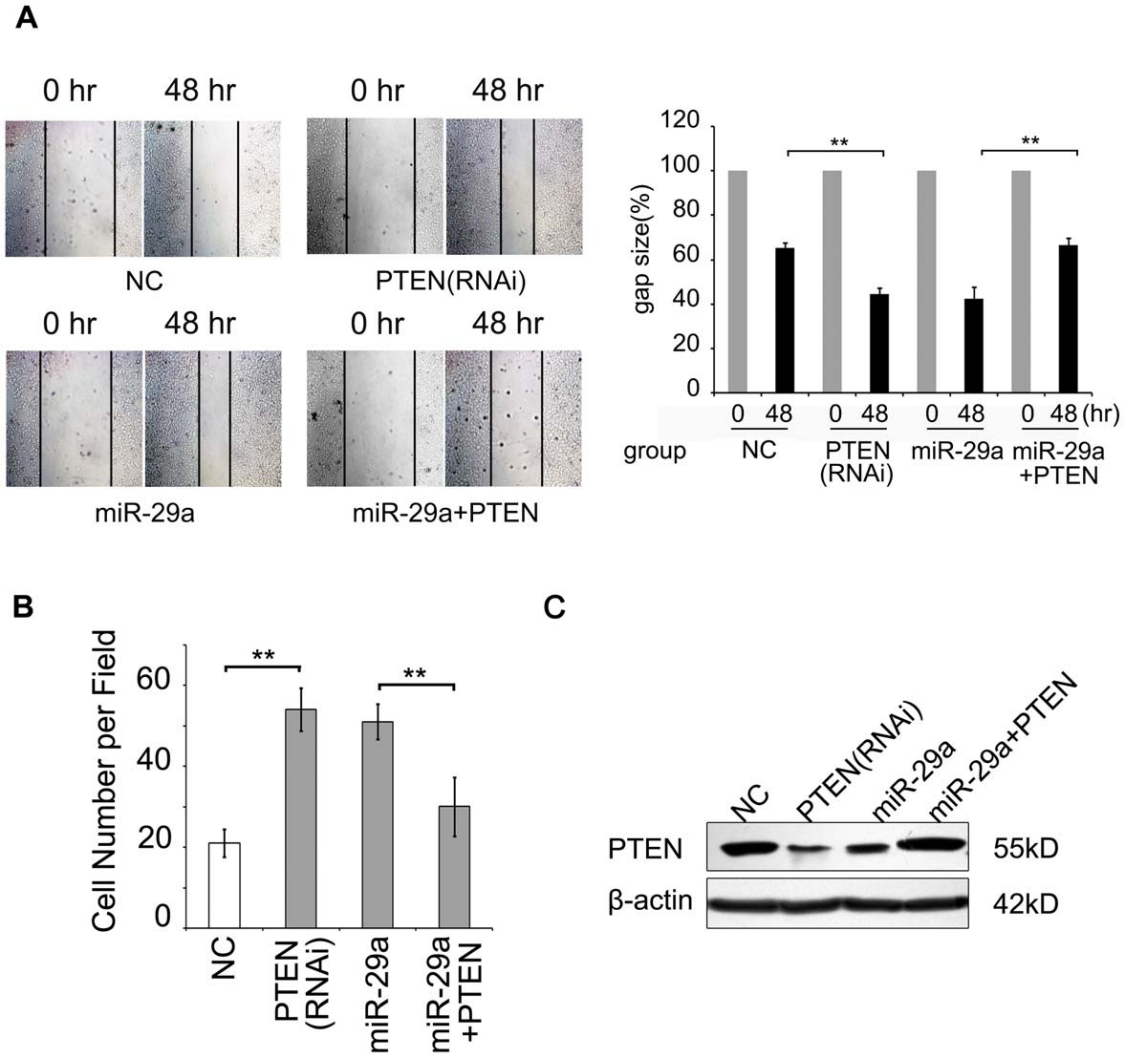
PTEN inhibits migration of tumor cells. (A) A wound healing assay was performed on HepG2 cells (1×10^6^, 6-cm plate) transfected with random siRNA, PTEN siRNA, pcDNA3-29a, and both pcDNA3-PTEN/pcDNA3-29a, respectively. One representative experiment is shown. Black arrows indicate the wound edge. (B) Modified Boyden's chamber assays showed that enhanced cell migration induced by miR-29a was reversed by the overexpression of PTEN in HepG2 cells. A histogram shows the relative cell number of five randomly selected fields. (C) The expression of PTEN was detected by western blot analysis in above transfected HepG2 cells. Statistically significant differences are indicated: *P<.05, **P<.01, Student's *t* test.

Accordingly, we identified that PTEN was a target gene of miR-29a. It has been reported that PTEN is able to inhibit the migration of hepatoma cells [21], [22]. To further understand the mechanism of miR-29a promoted migration, we examined the effect of PTEN on the migration ability of HepG2 cells mediated by miR-29a by knockdown the PTEN expression using siRNA. The wound healing and modified Boyden's chamber assays showed that downregulation of PTEN was able to promote the migration of HepG2 cells relative to control cells. Interestingly, the overexpression of PTEN was able to abolish the enhanced migration of HepG2 cells mediated by miR-29a by transient transfection with pcDNA3-PTEN plasmid (Fig. 4A and 4B). Western blot confirmed the expression of PTEN after the transfection (Fig. 4C). Therefore, our finding suggests miR-29a increases the migration of hepatoma cells through PTEN target gene.

### Akt phosphorylation is regulated by miR-29a through PTEN

**Figure 5:**
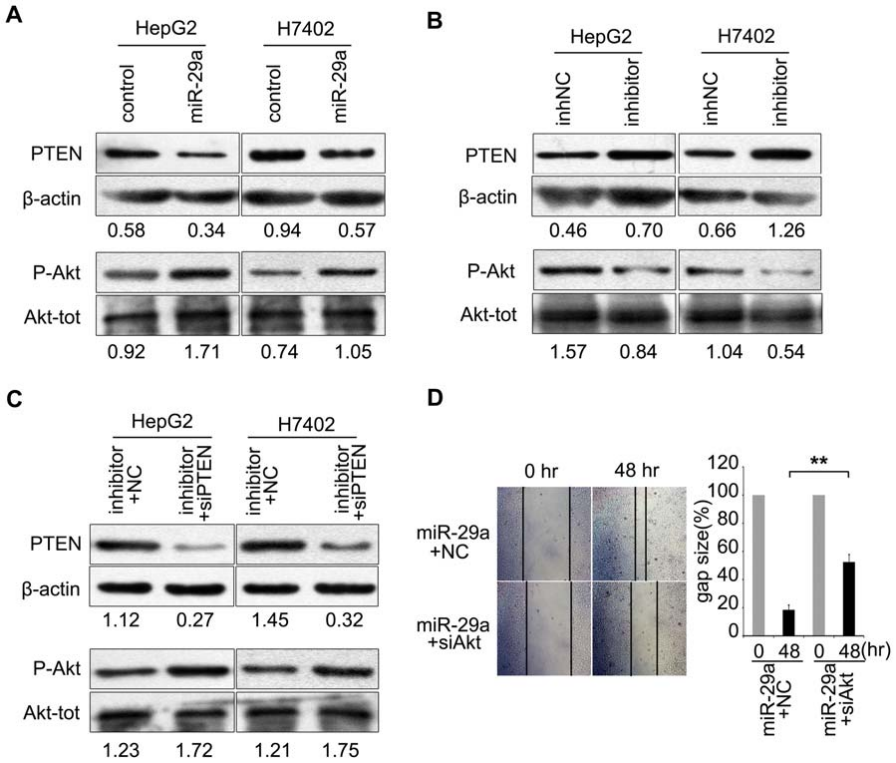
Akt phosphorylation is regulated by miR-29a through PTEN. (A, B) The phosphorylation level of Akt (P-Akt) and the expression level of total Akt (Akt-tot) were detected by Western blot analysis in HepG2 and H7402 cell lines after transfection of miR-29a (or a specific inhibitor). (C) Western blot analysis showed the phosphorylation of Akt (P-Akt) and the expression of total Akt (Akt-tot) in the cells treated by PTEN siRNA when the hepatoma cells were transfected with miR-29a inhibitor. Protein bands were quantified using Quantity One software (Bio-Rad). The value under each lane indicates the relative expression level of PTEN or phosphorylated Akt, which is represented by the intensity ratio between PTEN or phosphorylated Akt and β-actin or total Akt bands in each lane. (D) A wound healing assay showed that enhanced cell migration by miR-29a was abolished by Akt siRNA. One representative experiment is shown. Black arrows indicate the wound edge.

Protein kinase B (Akt) is a protein serine-threonine protein kinase involved in the regulation of cell growth, cell survival, and cell migration [19], [26]. Expression of PTEN leads to dephosphorylation of Akt and inhibition of cell migration. To confirm the role of miR-29a in regulation of PTEN, we next assessed the role of miR-29a on the expression of phosphorylated Akt, a downstream effector of PTEN. We found that the transfection with miR-29a in hepatoma cells led to the downregulation of PTEN and increased the level of serine phosphorylation of Akt at ser473 (Fig. 5A). Moreover, the inhibitor of miR-29a significantly reduced the phosphorylation of Akt in HCC cell lines (Fig. 5B). Meanwhile, we showed that the treatment with PTEN siRNA could abolish the inhibited phosphorylation of Akt mediated by miR-29a inhibitor (Fig. 5C). Interestingly, wound healing assay showed that knockdown of Akt by siRNA was able to abolish the enhanced migration ability of HepG2 cells mediated by miR-29a (Fig. 5D). The total Akt and the phosphorylation level of Akt were shown in Fig. S3A.

It has been reported that MMP-2 is one of targets of miR-29b in Hela cells and prostate cancer cells [27], [28], so that we are interested in the association of miR-29a and MMP-2. We assessed the effect of miR-29a on the expression of MMPs. Surprisingly, our data revealed that the transfection with miR-29a increased the expression level of MMP-2 mRNA in HCC cell lines by real time PCR (Fig. S3B and S3C). Whereas, the expression level of MMP-2 was decreased by the transfection with the miR-29a inhibitor in the cells (Fig. S3D), suggesting that miR-29a is able to upregulate MMP2.

## Discussion

HBx is a multifunctional oncoprotein which plays many roles in the development of HCC. HBx deregulates a wide variety of host genes related to cell proliferation, cell cycle progress, apoptosis, metastasis, protein degradation pathways and genetic stability [29]. Previous studies demonstrated that HBx is associated with HCC cells migration [4], [5], [6], [7]. Although these changes triggered by HBx are known to be involved in the acquisition of metastatic properties, the possible contribution of HBx to the development of HCC remains far unclear. Recent studies have shown that miRNAs play a fundamental role in the invasion and metastasis of HCC [21], thereby opening a novel avenue to investigate the molecular mechanism of HCC progression and to develop potential therapeutics against HCC. It is also reported that HBx is able to upregulate miR-143 through NF-κB, which can promote HCC metastasis in an athymic nude mouse model [30]. However, the effect of miRNAs has not been explored extensively in HBV-HCC.

In our present study, we first found that miR-29a level was upregulated in *p21-HBx* transgenic mice and varied significantly between HepG2 (or H7402) cells with or without HBx stable expression (Fig. 1A and 1C). Meanwhile, we found that miR-29a was upregulated in HepG2.2.15 cells that constitutively replicate HBV relative to HepG2 cells (Fig. 1D), which supports that HBx upregulates miR-29a. However, our data showed that miR-29a was upregulated in only 4 of the 11 clinical HCC samples (Fig. S1B). Previous study reported that miR-29c was downregulated in HBV/HCV positive HCC samples compared with normal tissues [31]. We can not answer the controversial issue, so that we consider that this can be partially explained by multifactorial etiology of cancer and the complexity of clinical tissues. For example, Ramiro Garzon et al. found that miR-29a was downregulated in acute myeloid leukemia (AML) patients [32]. While, Yoon-Chi Han, et al. reported that miR-29a was overexpressed in AML patients [14]. The similar phenomena were observed for miR-34a in HCC patients [33], [34]. In addition, Santanam U et al. reported that miR-29a was upregulated in indolent human B-cell chronic lymphocytic leukemia (B-CLL) as compared with aggressive B-CLL and normal CD19+ B cells [15]. Thus, we speculate that miR-29a may express at different stages of cancer development. Our finding suggests that the upregulation of miR-29a may be involved in the development of HCC.

In addition, we also observed that the expression level of miR-29a was higher in MHCC-97H cells compared with MHCC-97L cells (Fig. S2A). As elevated miR-29a expression was found in MHCC-97H which has high metastasis potential, we present a hypothesis that miR-29a may contribute to sustain a high migration ability mediated by HBx. Thus, we tested whether miR-29a has the ability to enhance the migration of tumor cells. We observed that miR-29a was able to promote the migration of hepatoma cells. Then, we found a significant reduce of migration after we added miR-29a inhibitor into HepG2-X cells (Fig. 2D and 2E). Thus, we conclude that HBx increases the migration of hepatoma cells through enhancing the expression of miR-29a, which is consistent with the previous report that miR-29a promote metastasis in breast cancer [13].

To better understand the underlying mechanism of miR-29a induced hepatoma cell migration, we found that PTEN was potentially a target gene of miR-29a using bioinformatics method (Fig. 3A). We subsequently confirmed that PTEN was a direct target of miR-29a, which was able to decrease PTEN expression at both mRNA and protein levels (Fig. 3B, 3C, and 3D). Therefore, PTEN is regulated by miR-29a besides miR-21 [21] and miR-221&222 [22]. We tried to detect the expression of miR-21 and miR-221 in HBx positive cells and in the *p21-HBx* transgenic mice. The result showed that the expression of miR-21 and miR-221 was not affected by HBx. Previous study also showed that HBx was able to downregulate PTEN expression in liver cells [25], which is consistent with our report. Importantly, we found that PETN was able to abolish the promoted migration of HepG2 cells mediated by miR-29a (Fig. 4A and 4B), suggesting that miR-29a increases the migration of tumor cells by PTEN target gene.

To demonstrate the role of miR-29a in regulation of PTEN, we further examined the effect of miR-29a on the phosphorylation level of Akt, a downstream target of PTEN. Interestingly, our data showed that miR-29a was able to promote the phosphorylation of Akt (Fig. 5A and 5B), supporting that miR-29a regulates PTEN. In function, we showed that knockdown of Akt expression abolished the increased migration ability mediated by miR-29a in HepG2 cells (Fig. 5D). It further supports that miR-29a promotes cell migration through PTEN. Meanwhile, our data showed that miR-29a upregulated the expression of MMP-2 (Fig. S3B and S3C). However, previous studies reported that miR-29a targeted p85α (the regulatory subunit of PI3 kinase), which may result in the reduced phosphorylation of Akt [35]. It has also been reported that MMP-2 is one of miR-29b targets in Hela cells and prostate cancer cells [27], [28]. One possible explanation is that it is due to a context-dependent pattern for miR-29a in tumorigenicity. Many studies demonstrated that a miRNA could be considered either as a tumor suppressor or as an oncogene depending on its targets in different tissues and cell types [9], [36], [37]. A recent study reported that microRNA-340-mediated degradation of microphthalmia-associated transcription factor mRNA was inhibited by the coding region determinant-binding protein, which provided an important mechanism that the interaction of miRNA and its target genes could be prevented, resulting in stabilization of the target gene transcript and elevated expression and transcriptional activity [38]. Thus, our findings suggest that miR-29a promotes hepatoma cell migration through targeting PTEN involving the activation of phosphorylated Akt and MMP-2.

Taken together, here we report that miR-29a is able to directly regulate PTEN in the promotion of hepatoma cell migration mediated by HBx. Thus, our finding provides new insights into the regulation of altered miRNA expression in contributing to the tumor phenotype.

## Materials and Methods

### Cell culture

HepG2, H7402, HepG2.2.15, MHCC-97L, MHCC-97H cells were maintained in Dulbecco's modified Eagle's medium (Life Technologies, Inc., Gaithersburg, MD) supplemented with 10% fetal bovine serum (Life Technologies). The engineered cells of HepG2-X/H7402-X (stably transfected with the pCMV-HBx plasmid), and HepG2-P/H7402-P (stably transfected with the empty pcDNA3.0 vector plasmid) were generated in HepG2 and H7402 cells, respectively, using the Lipofectamine 2000 protocol (Invitrogen, USA) as previously described [23].

### Patient samples

The 11 clinical HCC tissues and the corresponding nearby noncancerous livers used in this study were obtained from patients who underwent radical resection at Tianjin First Center Hospital (Tianjin, China). Tissues were snap frozen in liquid nitrogen. Written consent approving the use of their tissues for research purposes after operation was obtained from each patient. The study was approved by the Institute Research Ethics Committee at the Nankai University.

### RNA extraction and quantitative reverse transcription PCR analysis (qRT-PCR)

Extraction of total RNA of the cells (or aged 6 month liver tissues from *p21-HBx* transgenic mice [3]) and reverse transcription were carried out as described previously [39]. For tissues RNA extraction, grind frozen tissues to a fine powder using a mortar and pestle under liquid nitrogen quickly and then treated as cells. To quantify mature miRNA expression, the total RNA was isolated by a modified Trizol protocol. Total RNA was polyadenylated by poly (A) polymerase (Ambion, Austin, TX). A 50 µl polyadenylation reaction was generated using 10 µg total RNA and 1 µl (2U) poly (A) polymerase according to the manufacturer's protocol. The reaction was incubated at 37°C for 60 min after which poly (A)-tailed total RNA was recovered by phenol/chloroform extraction and ethanol precipitation. Reverse transcription was performed using 1 µg poly (A)-tailed total RNA, and 1 µl ImPro-II Reverse Transcriptase (Promega, USA) according to manufacturer's instructions. Quantitative real time PCR was performed using the quantitative SYBR Green PCR kit (TaKaRa Bio, China). One primer is microRNA-specific (Table S1), and the other is a universal primer (Table S1). U6 small nuclear RNA and GAPDH were used for normalization (Table S1). To examine the specificity of the qRT-PCR, the dissociation curve analysis is performed after a completed PCR. Primers used are listed in Table S1.

### RNA interference

pSilencer-HBx was used to produce small interfering RNAs (siRNAs) targeting HBx mRNA, and pSilencer-control was used as negative control [40]. MiR-29a inhibitor, inhibitor negative control (inhNC), siRNA duplexes target human PTEN and total Akt [41] were synthesized and purified by RiboBio (Guangzhou, China). SiRNA duplexes with non-specific sequences were used as siRNA negative control (NC). Different siRNAs were transfected separately into cells by using Lipofecatmine 2000 (Invitrogen) reagent and medium was replaced 6h after transfection.

### Construction of plasmids

To construct a plasmid expressing miR-29a, we amplified a 263 bp DNA fragment containing miR-29a precursor from HepG2 genomic DNA (Table S1). The amplified fragment was cloned into a pcDNA3.0 vector, which was termed pcDNA3-29a. The full-length human PTEN expression vector was amplified from HepG2 cDNA (Table S1), and then cloned into a pcDNA3.0 vector, which was termed pcDNA3-PTEN. An empty pcDNA3.0 vector was used as control (control). To construct 3′UTR reporter plasmids, two ∼400 bp fragments of the 3′UTR of human PTEN containing the putative miR-29a binding site were amplified from HepG2 cells cDNA, and cloned downstream of the luciferase reporter gene of a pGL3-control vector (Promega). To introduce four base pair deletion of the seed region of the miR-29a binding site, we used a PCR approach where the seeds sequences were deleted in the primers used for PCR reactions (Table S1).

### Dual-luciferase reporter gene assay

HepG2 cells were seeded in 24-well plates. After 24 h, cells were co-transfected with pGL3 control vector containing the 3′UTR fragment of PTEN, Renilla vector (pRL-TK) and pcDNA3-29a or pcDNA3.0 empty plasmid. Luciferase activities were measured 36 h post-transfection using the Dual-Luciferase Reporter Assay System (Promega). Luciferase activity was normalized for transfection efficiency using the corresponding Renilla luciferase activity. All experiments were performed at least three times.

### Western blot analysis

Cells were washed in PBS, and cellular proteins were extracted in RIPA buffer (Biomed, China) for 30 min at 4°C. Lysates were cleared by centrifugation, and proteins were separated by gel electrophoresis. Membranes were blocked in PBS-0.1% Tween20 (PBS-T)/5% (w/v) milk for 1 hr at room temperature. Membranes were then incubated with primary antibodies diluted in PBS-T for 2 hr at room temperature. Subsequently, membranes were washed with PBS-T and incubated with peroxidase-conjugated secondary antibody diluted in PBS-T at room temperature for 1 hr. Membranes were washed in PBS-T and bound antibody was detected by enhanced chemiluminescence system Western Blotting Detection Reagents (Amersham Biosciences, Buckinghamshire, UK). After 48 hr transfection, Western blot analysis was performed as above; the primary antibodies were anti-PTEN (Santa Cruz, USA), anti-HBx (Abcam, Cambridge, UK), anti-Akt (Santa Cruz), anti-phosphorylated Akt (Cell Signaling, USA) and anti-β-actin (Sigma, USA). All experiments were repeated 3 times. Protein bands were quantified using Quantity One software (Bio-Rad).

### Wound healing assay

HepG2 cells seeded at 2×10^5^ in 2 ml of Dulbecco's modified Eagle's medium were cultured overnight at 37°C in 6-well plates. After 24 hr, transfected cells were wounded by dragging a 1 ml pipette tip through the monolayer. Cells were washed using PBS to remove cellular debris and allowed to migrate for 48 hr. Wound closure or cell migration images were photographed when the scrape wound was introduced (0 hr) and at a designated time (48 hr) after wounding using an inverted microscope. The relative surface traveled by the leading edge was assessed using Motic Images Advanced 3.2 software. The individual gaps were measured at each time point, and the speed of migration was acquired by dividing the length of the gap by the culture time. Three replicates each of two independent experiments were performed.

### Modified boyden's chamber assay

Cell migration was assessed using a modified Boyden's chamber method as described previously [42]. Briefly, 96-well Boyden's chamber was used to measure cell migratory ability and a polycarbonate membrane with 8-µm pores was used in these experiments. Cells were resuspended in serum-free DMEM medium containing 0.2% BSA and were loaded into the upper wells of the chamber. The lower wells were filled with DMEM medium containing 10% fetal calf serum as chemoattractant for cell migration. Cells adhering to the lower membrane were counted through an inverted microscope.

### Statistical analysis

All data were expressed as mean ± SD. Statistical analysis was performed by the Student's *t* test. P<.05 was indicated to be statistical significant. Association between expression levels of miR-29a and PTEN in tissues was explored using Pearson′s correlation coefficient.

## Supporting Information

Figure S1 (A) The relative mRNA expression levels of HBx in clinical HBV-positive HCC tissues were examined by qRT-PCR. (B) The relative expression of miR-29a in clinical HBV-positive HCC tissues was examined by qRT-PCR. Statistically significant differences are indicated: *P<.05, **P<.01 (Student's *t* test).
(TIF)

Figure S2 (A) The expression of miR-29a in MHCC-97H and MHCC-97L cells was examined by qRT-PCR. (B) Correlation between miR-29a and PTEN levels in clinical HCC tissues was analyzed. (C) The expression levels of HBx and PTEN were detected by Western blot analysis in HepG2, HepG2-P and HepG2-X (or H7402, H7402-P and H7402-X) cells, respectively. (D) The expression levels of HBx and PTEN were detected by Western blot in HepG2-X (or H7402-X) cells after HBx knockdown by RNAi. (E) The expression of PTEN was detected by Western blot in MHCC-97L and MHCC-97H cells.
(TIF)

Figure S3 (A) The efficiency of Akt siRNA was detected by Western blot. (B, C) The expression levels of MMP-1, MMP-2, MMP-3, MMP-9 and MMP-11 were examined in HepG2 and H7402 cells transfected with miR-29a by qRT-PCR. (D) The expression level of MMP-2 mRNA was assessed in HepG2 and H7402 cells transfected with a specific miR-29a inhibitor by qRT-PCR. Statistically significant differences are indicated: *P<.05, **P<.01 (Student's *t* test).
(TIF)

Table S1 **List of primers used in this paper.**
(DOC)

Table S2 **The characteristics of patients.**
(DOC)

## References

[1] McGlynn KA, London WT (2005). Epidemiology and natural history of hepatocellular carcinoma.. Best Pract Res Clin Gastroenterol.

[2] Zhang X, Zhang H, Ye L (2006). Effects of hepatitis B virus X protein on the development of liver cancer.. J Lab Clin Med.

[3] Wang Y, Cui F, Lv Y, Li C, Xu X (2004). HBsAg and HBx knocked into the p21 locus causes hepatocellular carcinoma in mice.. Hepatology.

[4] Lara-Pezzi E, Majano PL, Yanez-Mo M, Gomez-Gonzalo M, Carretero M (2001). Effect of the hepatitis B virus HBx protein on integrin-mediated adhesion to and migration on extracellular matrix.. Journal of Hepatology.

[5] Xia LM, Huang WJ, Wu JG, Yang YB, Zhang Q (2009). HBx protein induces expression of MIG and increases migration of leukocytes through activation of NF-kappaB.. Virology.

[6] Chung TW, Lee YC, Kim CH (2004). Hepatitis B viral HBx induces matrix metalloproteinase-9 gene expression through activation of ERK and PI-3K/AKT pathways: involvement of invasive potential.. Faseb Journal.

[7] Zhang F, Wang Q, Ye L, Feng Y, Zhang X (2010). Hepatitis B virus X protein upregulates expression of calpain small subunit 1 via nuclear factor-kappaB/p65 in hepatoma cells.. J Med Virol.

[8] Filipowicz W, Bhattacharyya SN, Sonenberg N (2008). Mechanisms of post-transcriptional regulation by microRNAs: are the answers in sight?. Nature Reviews Genetics.

[9] Chen CZ (2005). MicroRNAs as oncogenes and tumor suppressors.. N Engl J Med.

[10] Lu J, Getz G, Miska EA, Alvarez-Saavedra E, Lamb J (2005). MicroRNA expression profiles classify human cancers.. Nature.

[11] Pekarsky Y, Santanam U, Cimmino A, Palamarchuk A, Efanov A (2006). Tcl1 expression in chronic lymphocytic leukemia is regulated by miR-29 and miR-181.. Cancer Res.

[12] Fabbri M, Garzon R, Cimmino A, Liu Z, Zanesi N (2007). MicroRNA-29 family reverts aberrant methylation in lung cancer by targeting DNA methyltransferases 3A and 3B.. Proc Natl Acad Sci U S A.

[13] Gebeshuber CA, Zatloukal K, Martinez J (2009). miR-29a suppresses tristetraprolin, which is a regulator of epithelial polarity and metastasis.. EMBO Rep.

[14] Han YC, Park CY, Bhagat G, Zhang J, Wang Y (2010). microRNA-29a induces aberrant self-renewal capacity in hematopoietic progenitors, biased myeloid development, and acute myeloid leukemia.. J Exp Med.

[15] Santanam U, Zanesi N, Efanov A, Costinean S, Palamarchuk A (2010). Chronic lymphocytic leukemia modeled in mouse by targeted miR-29 expression.. Proc Natl Acad Sci U S A.

[16] Li DM, Sun H (1997). TEP1, encoded by a candidate tumor suppressor locus, is a novel protein tyrosine phosphatase regulated by transforming growth factor beta.. Cancer Res.

[17] Li J, Yen C, Liaw D, Podsypanina K, Bose S (1997). PTEN, a putative protein tyrosine phosphatase gene mutated in human brain, breast, and prostate cancer.. Science.

[18] Wan XW, Jiang M, Cao HF, He YQ, Liu SQ (2003). The alteration of PTEN tumor suppressor expression and its association with the histopathological features of human primary hepatocellular carcinoma.. J Cancer Res Clin Oncol.

[19] Dasari VR, Kaur K, Velpula KK, Gujrati M, Fassett D (2010). Upregulation of PTEN in glioma cells by cord blood mesenchymal stem cells inhibits migration via downregulation of the PI3K/Akt pathway.. PLoS One.

[20] Dey N, Crosswell HE, De P, Parsons R, Peng Q (2008). The protein phosphatase activity of PTEN regulates SRC family kinases and controls glioma migration.. Cancer Res.

[21] Meng F, Henson R, Wehbe-Janek H, Ghoshal K, Jacob ST (2007). MicroRNA-21 regulates expression of the PTEN tumor suppressor gene in human hepatocellular cancer.. Gastroenterology.

[22] Garofalo M, Di Leva G, Romano G, Nuovo G, Suh SS (2009). miR-221&222 regulate TRAIL resistance and enhance tumorigenicity through PTEN and TIMP3 downregulation.. Cancer Cell.

[23] Wang Q, Zhang W, Liu Q, Zhang X, Lv N (2010). A mutant of hepatitis B virus X protein (HBxDelta127) promotes cell growth through a positive feedback loop involving 5-lipoxygenase and fatty acid synthase.. Neoplasia.

[24] Li Y, Tang ZY, Ye SL, Liu YK, Chen J (2001). Establishment of cell clones with different metastatic potential from the metastatic hepatocellular carcinoma cell line MHCC97.. World J Gastroenterol.

[25] Chung TW, Lee YC, Ko JH, Kim CH (2003). Hepatitis B Virus X protein modulates the expression of PTEN by inhibiting the function of p53, a transcriptional activator in liver cells.. Cancer Res.

[26] Dudek H, Datta SR, Franke TF, Birnbaum MJ, Yao R (1997). Regulation of neuronal survival by the serine-threonine protein kinase Akt.. Science.

[27] Liu Y, Taylor NE, Lu L, Usa K, Cowley AW (2010). Renal medullary microRNAs in Dahl salt-sensitive rats: miR-29b regulates several collagens and related genes.. Hypertension.

[28] Steele R, Mott JL, Ray RB (2010). MBP-1 upregulates miR-29b that represses Mcl-1, collagens, and matrix-metalloproteinase-2 in prostate cancer cells.. Genes Cancer.

[29] Zhang WY, Cai N, Ye LH, Zhang XD (2009). Transformation of human liver L-O2 cells mediated by stable HBx transfection.. Acta Pharmacol Sin.

[30] Zhang X, Liu S, Hu T, He Y, Sun S (2009). Up-regulated microRNA-143 transcribed by nuclear factor kappa B enhances hepatocarcinoma metastasis by repressing fibronectin expression.. Hepatology.

[31] Ura S, Honda M, Yamashita T, Ueda T, Takatori H (2009). Differential microRNA expression between hepatitis B and hepatitis C leading disease progression to hepatocellular carcinoma.. Hepatology.

[32] Garzon R, Heaphy CE, Havelange V, Fabbri M, Volinia S (2009). MicroRNA 29b functions in acute myeloid leukemia.. Blood.

[33] Pineau P, Volinia S, McJunkin K, Marchio A, Battiston C (2010). miR-221 overexpression contributes to liver tumorigenesis.. Proc Natl Acad Sci U S A.

[34] Li N, Fu H, Tie Y, Hu Z, Kong W (2009). miR-34a inhibits migration and invasion by down-regulation of c-Met expression in human hepatocellular carcinoma cells.. Cancer Lett.

[35] Park SY, Lee JH, Ha M, Nam JW, Kim VN (2009). miR-29 miRNAs activate p53 by targeting p85 alpha and CDC42.. Nat Struct Mol Biol.

[36] Gaur A, Jewell DA, Liang Y, Ridzon D, Moore JH (2007). Characterization of microRNA expression levels and their biological correlates in human cancer cell lines.. Cancer Res.

[37] Calin GA, Croce CM (2006). MicroRNA signatures in human cancers.. Nat Rev Cancer.

[38] Goswami S, Tarapore RS, Teslaa JJ, Grinblat Y, Setaluri V (2010). MicroRNA-340-mediated degradation of microphthalmia-associated transcription factor mRNA is inhibited by the coding region determinant-binding protein.. J Biol Chem.

[39] Qin X, Zhang H, Zhou X, Wang C, Zhang X (2007). Proliferation and migration mediated by Dkk-1/Wnt/beta-catenin cascade in a model of hepatocellular carcinoma cells.. Transl Res.

[40] Zhang X, Dong N, Yin L, Cai N, Ma H (2005). Hepatitis B virus X protein upregulates survivin expression in hepatoma tissues.. J Med Virol.

[41] Katome T, Obata T, Matsushima R, Masuyama N, Cantley LC (2003). Use of RNA interference-mediated gene silencing and adenoviral overexpression to elucidate the roles of AKT/protein kinase B isoforms in insulin actions.. J Biol Chem.

[42] You J, Mi D, Zhou X, Qiao L, Zhang H (2009). A positive feedback between activated extracellularly regulated kinase and cyclooxygenase/lipoxygenase maintains proliferation and migration of breast cancer cells.. Endocrinology.

