# Supplementary figures and images for: Upregulated MicroRNA-29a by Hepatitis B Virus X Protein Enhances Hepatoma Cell Migration by Targeting PTEN in Cell Culture Model
Source: PLoS One. 2011 May 5;6(5):e19518. doi: 10.1371/journal.pone.0019518 (PMC3088678; doi:10.1371/journal.pone.0019518)

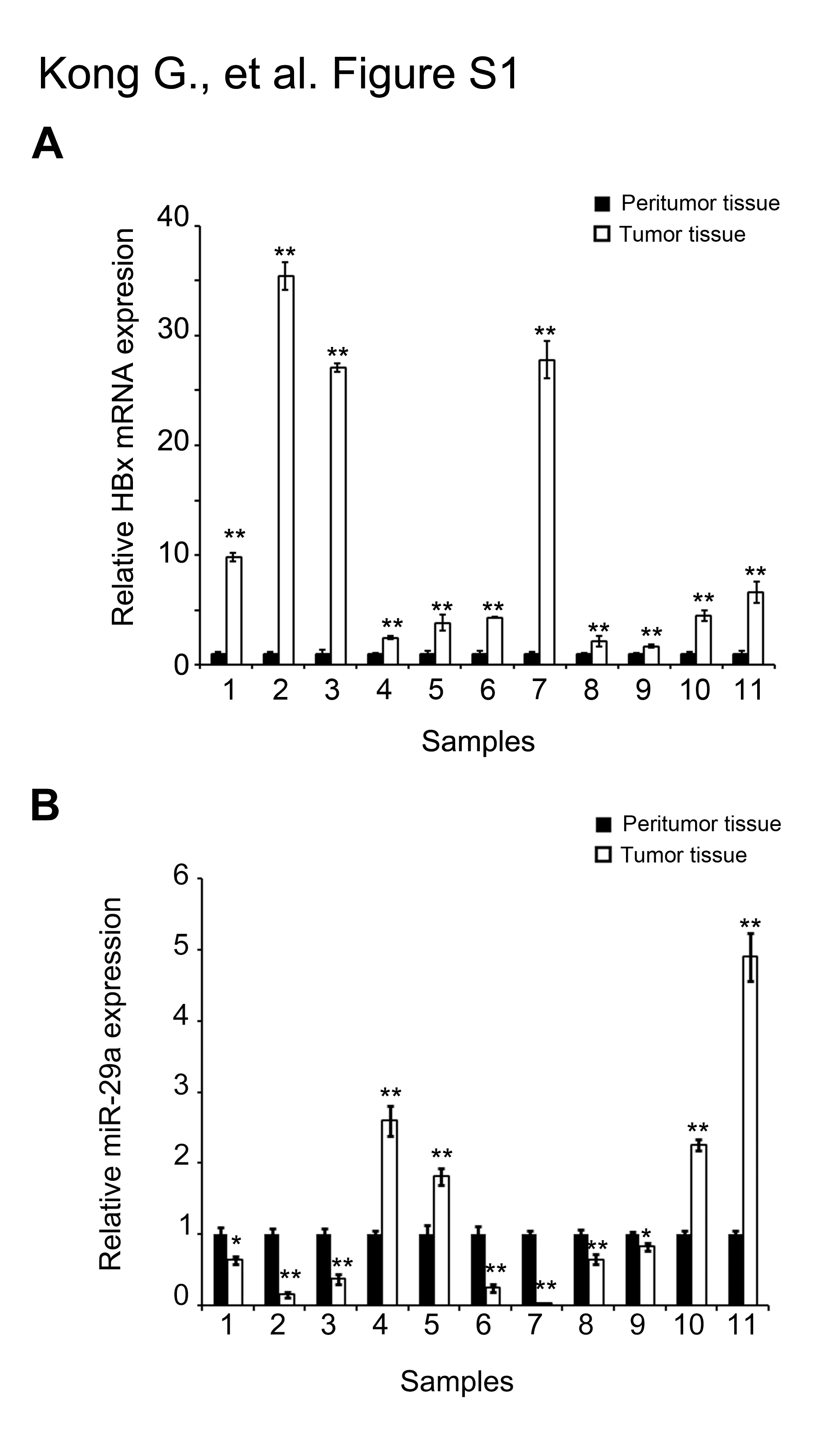

Supplement: Figure S1 — (A) The relative mRNA expression levels of HBx in clinical HBV-positive HCC tissues were examined by qRT-PCR. (B) The relative expression of miR-29a in clinical HBV-positive HCC tissues was examined by qRT-PCR. Statistically significant differences are indicated: *P<.05, **P<.01 (Student's t test). (TIF) [file pone.0019518.s001.tif]

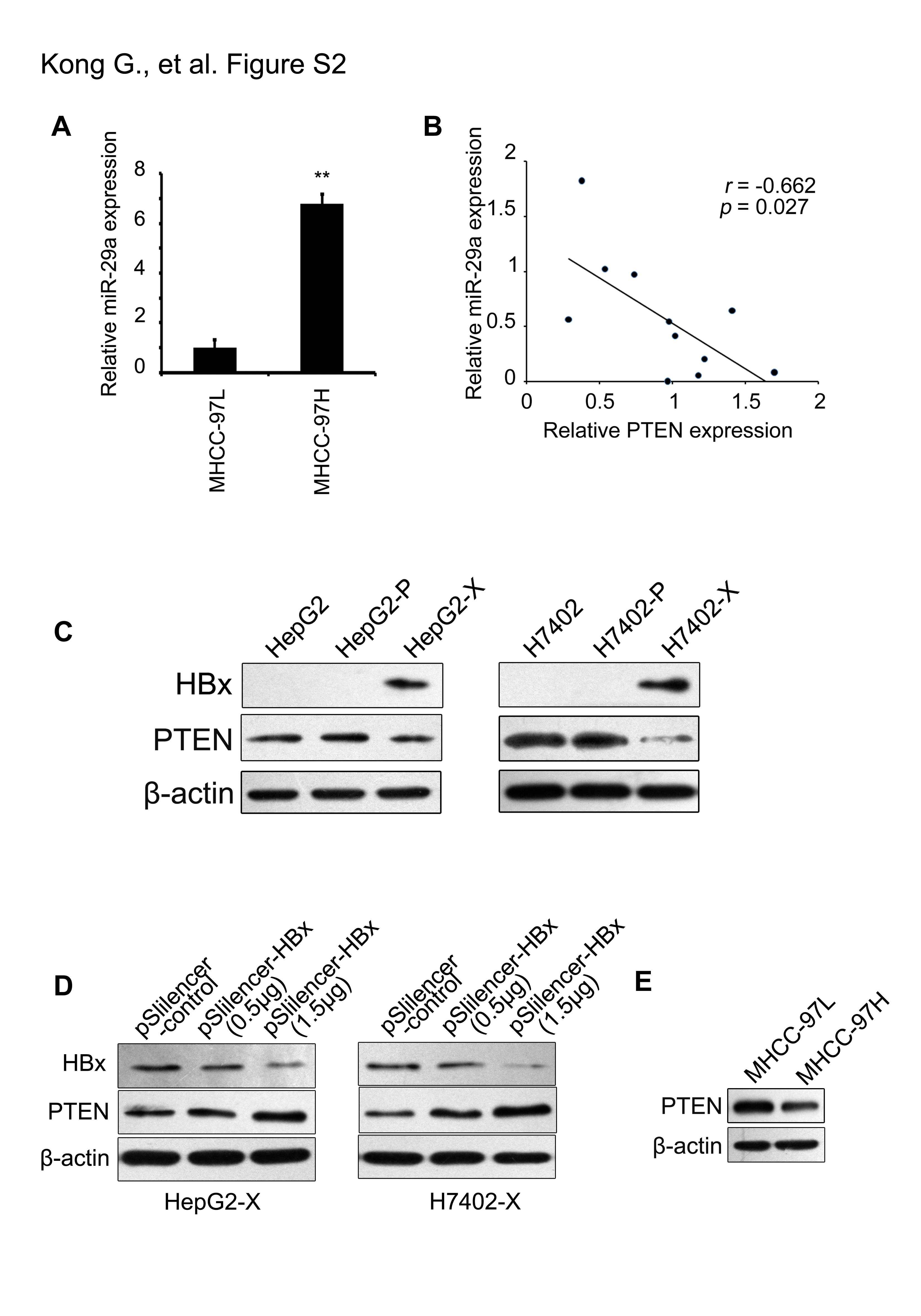

Supplement: Figure S2 — (A) The expression of miR-29a in MHCC-97H and MHCC-97L cells was examined by qRT-PCR. (B) Correlation between miR-29a and PTEN levels in clinical HCC tissues was analyzed. (C) The expression levels of HBx and PTEN were detected by Western blot analysis in HepG2, HepG2-P and HepG2-X (or H7402, H7402-P and H7402-X) cells, respectively. (D) The expression levels of HBx and PTEN were detected by Western blot in HepG2-X (or H7402-X) cells after HBx knockdown by RNAi. (E) The expression of PTEN was detected by Western blot in MHCC-97L and MHCC-97H cells. (TIF) [file pone.0019518.s002.tif]

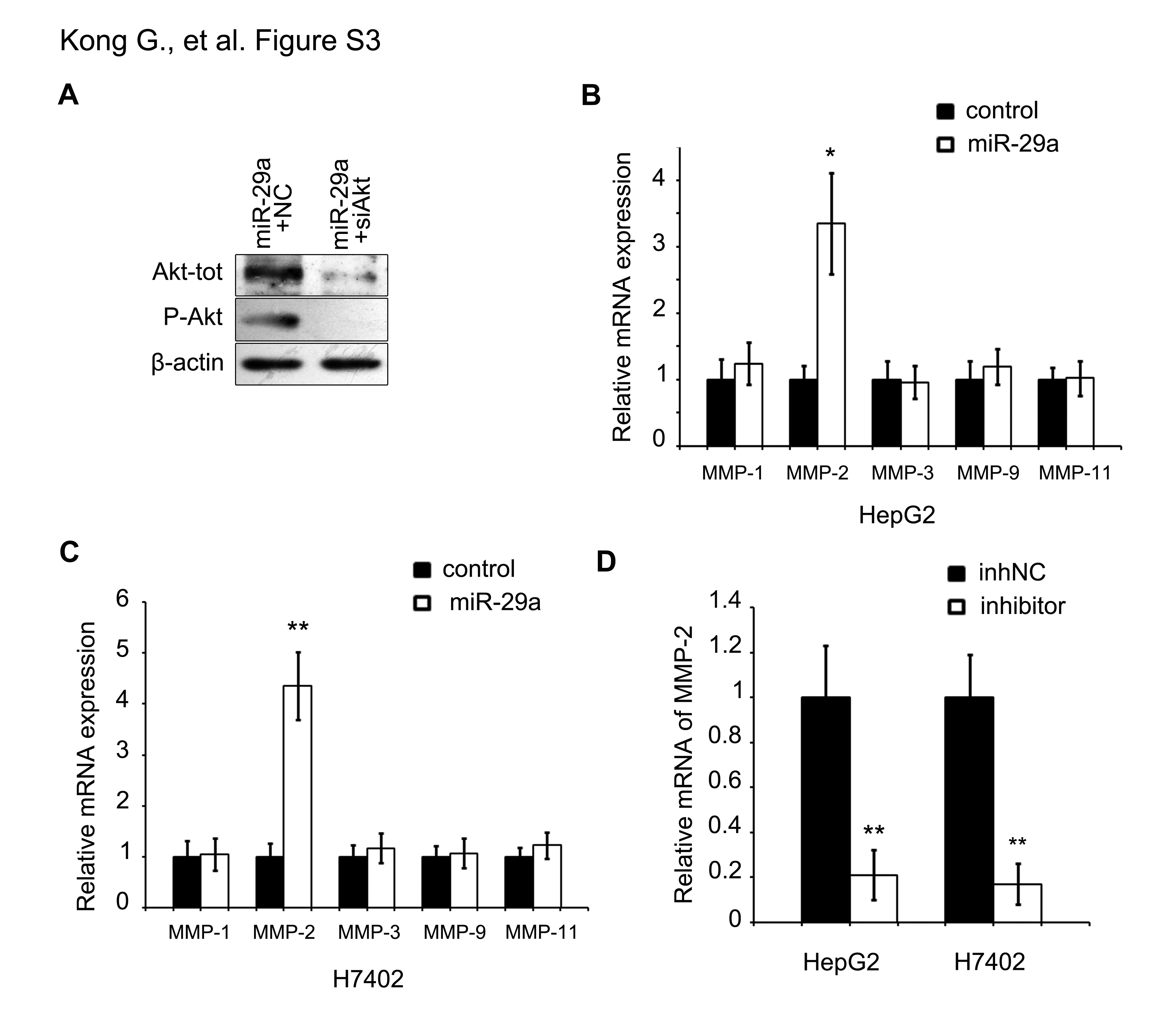

Supplement: Figure S3 — (A) The efficiency of Akt siRNA was detected by Western blot. (B, C) The expression levels of MMP-1, MMP-2, MMP-3, MMP-9 and MMP-11 were examined in HepG2 and H7402 cells transfected with miR-29a by qRT-PCR. (D) The expression level of MMP-2 mRNA was assessed in HepG2 and H7402 cells transfected with a specific miR-29a inhibitor by qRT-PCR. Statistically significant differences are indicated: *P<.05, **P<.01 (Student's t test). (TIF) [file pone.0019518.s003.tif]
