# Supplementary material for: Upregulated MicroRNA-29a by Hepatitis B Virus X Protein Enhances Hepatoma Cell Migration by Targeting PTEN in Cell Culture Model
Source: PLoS One. 2011 May 5;6(5):e19518. doi: 10.1371/journal.pone.0019518 (PMC3088678; doi:10.1371/journal.pone.0019518)
Supplement: Table S1 — List of primers used in this paper. (DOC) [file pone.0019518.s004.doc]

**Table S1.** List of primers used in this paper.

| **Gene** | **Primer** | **Sequence(5′-3′)** |
| --- | --- | --- |
| **Primers for Cloning** | |  |
| miR-29a | forward | CCCAAGCTTATGCTGGATTTAGTAAGA |
|  | reverse | CCGCTCGAGAACAGGGCAAATAAATGCA |
| PTEN | forward | CCGGAATTCATGACAGCCATCATCAAAGAGATCG |
|  | reverse | CCGCTCGAGTCAGACTTTTGTAATTTGTGTATGC |
| PTEN 3’UTR-1 | forward | CGTTCTAGAGCATGTATTCGGGTTAGG |
|  | reverse | GGGGGCCGGCCAAGCCCATTCTTTGTTGA |
| PTEN 3’UTR-2 | forward | CGTTCTAGATTTAAGCGGAGTACAACT |
|  | reverse | GGGGGCCGGCCTGAATGAAACTGACAAGG |
| PTEN 3’UTR-1-del | forward | TAAAGCATATGCTAGAAAAGGCAG |
|  | reverse | CTGCCTTTTCTAGCATATGCTTTA |
| PTEN 3’UTR-2-del | forward | TTCAATAACTTATGCTGAAATTGTTCA |
|  | reverse | TGAACAATTTCAGCATAAGTTATTGAA |
| **Primers for qRT-PCR** | |  |
| PTEN | forward | GCATGTATTCGGGTTAGG |
|  | reverse | TGAATGAAACTGACAAGG |
| HBx | forward | ATGGCTGCTAGGGTGTGCTG |
|  | reverse | CTAGGCAGAGGTGAAAAAGTTGC |
| GAPDH | forward | CATCACCATCTTCCAGGAGCG |
|  | reverse | TGACCTTGCCCACAGCCTTG |
| MMP-1 | forward | AGCTAGCTCAGGATGACATTGATG |
|  | reverse | GCCGATGGGCTGGACAG |
| MMP-2 | forward | TGGCGATGGATACCCCTTT |
|  | reverse | TTCTCCCAAGGTCCATAGCTCAT |
| MMP-3 | forward | TGGCATTCAGTCCCTCTATGG |
|  | reverse | AGGACAAAGCAGGATCACAGTT |
| MMP-9 | forward | CCTGGGCAGATTCCAAACCT |
|  | reverse | GCAAGTCTTCCGAGTAGTTTTGGA |
| MMP-11 | forward | TGACTTCTTTGGCTGTGCC |
|  | reverse | GTTGTCATGGTGGTTGTACCC |
| miR-29a | forward | TAGCACCATCTGAAATCGGTTA |
| miR-29c | forward | TAGCACCATTTGAAATCGGTTA |
|  | reverse | GCGAGCACAGAATTAATACGAC |
| U6 | forward | AGAGCCTGTGGTGTCCG |
|  | reverse | CATCTTCAAAGCACTTCCCT |
| **siRNA Duplexes** | |  |
| siPTEN | sense | GGCGCUAUGUGUAUUAUUAdTdT |
|  | antisense | UAAUAAUACACAUAGCGCCdTdT |
| siAkt | sense | UGCCCUUCUACAACCAGGAdTdT |
|  | antisense | UCCUGGUUGUAGAAGGGCAdTdT |
| NC | sense | UUCUCCGAACGUGUCACGUdTdT |
