# Supplementary material for: Upregulated MicroRNA-29a by Hepatitis B Virus X Protein Enhances Hepatoma Cell Migration by Targeting PTEN in Cell Culture Model
Source: PLoS One. 2011 May 5;6(5):e19518. doi: 10.1371/journal.pone.0019518 (PMC3088678; doi:10.1371/journal.pone.0019518)
Supplement: Table S2 — The characteristics of patients. (DOC) [file pone.0019518.s005.doc]

**Table S2.** The characteristics of patients

| Case No. | Age (yr) | Gender | Edmondson Grade | HBsAg |
| --- | --- | --- | --- | --- |
| 1 | 38 | M | III | + |
| 2 | 59 | M | III | + |
| 3 | 54 | M | III | + |
| 4 | 31 | M | III | + |
| 5 | 42 | F | II | + |
| 6 | 46 | M | II | + |
| 7 | 60 | M | II-III | + |
| 8 | 59 | M | III | + |
| 9 | 57 | F | II-III | + |
| 10 | 51 | M | II-III | + |
| 11 | 38 | M | II | + |
|  | | | |  |
